# Typho-Malaria

**Published:** 1898-03

**Authors:** 


TYPHO-MALARIA.—Is there a typho-malarial fever? Yes, in the brains of the doctor, but not in the bodies of the patients. There is no combined hybrid disease and it is only due to Woodward to say that he did not recognize a hybrid disease. Typho-malaria is a villainous name and should be banished from our vocabularies and no doctor should ever use it, particularly to his patients. It gives a man a wrong sense of security and the doctor wastes a lot of good medicine, a lot of quinine, for instance, because he thinks there is some symptom that points to malaria. I am happy to say that cases of typho-malaria are disappearing slowly from the health reports; they ought to be banished entirely. Chills, as I told you, occur frequently at the outset of a disease, and they may occur throughout the course of a typhoid fever. The state boards of health should hereafter return to every physician who sends in a diagnosis of typho-malaria his blank and ask for something better. It is too late in the day, gentlemen, to make that diagnosis.—Dr. William Osler, in *Maryland Medical Journal.*

In a paper read at Belton before the Texas Medical Association, in 1884, Dr. S. H. Stout, now of Dallas, took exactly the position now held by Prof. Osler.

---

Dr. R. H. L. Bibb, surgeon in chief of the Mexican National railroad, has resigned the position of surgeon to the American Hospital in Mexico City and removed to Saltillo, his former home. The reason for this sacrifice of a part of his personal interests is, that the climate of Mexico City does not agree with Mrs. Bibb and their young son. The doctor has tried several times to have them live in the city, and each time they fell into a decline, and it became absolutely necessary, as they could not go to him, for him to go to them. However, as Dr. Bibb formerly resided and practiced at Saltillo, where his reputation as a surgeon caused him to be sent for, hundreds of miles, there is little doubt that he can at once resume a lucrative practice there that will more than compensate for the loss of his position as surgeon of the American Hospital. The doctor is still on the staff of the Journal, and will shortly favor us with a contribution.

